# Current and future diagnostics of congenital heart disease (CHD)

**DOI:** 10.1515/medgen-2025-2008

**Published:** 2025-04-08

**Authors:** Chiara Vey, Nico Melnik, Gregor Dombrowsky, Marc-Phillip Hitz

**Affiliations:** Carl von Ossietzky University Institute of Medical Genetics Rahel-Straus-Str. 10 26133 Oldenburg Germany; Carl von Ossietzky University Institute of Medical Genetics Rahel-Straus-Str. 10 26133 Oldenburg Germany; Carl von Ossietzky University Institute of Medical Genetics Rahel-Straus-Str. 10 26133 Oldenburg Germany; Carl von Ossietzky University Institute of Medical Genetics Rahel-Straus-Str. 10 26133 Oldenburg Germany

## Abstract

Congenital heart defects (CHD) are one of the most common anomalies found among live births and represent a complex multifactorial condition. Given that more than 90 % of cases survive due to improved early treatment options (e.g., catheter intervention, surgical procedure, and improved intensive care), genotype-informed patient follow-up should consider lifelong treatment considering different types of comorbidities. Unfortunately, a thorough genetic workup is only offered to a minority of CHD patients. However, a comprehensive understanding of the genetic underpinnings combined with in-depth phenotyping would strengthen our knowledge regarding the impact of environmental (e.g., pre-gestational diabetes) and genetic causes ranging from aneuploidies to single variants and more complex inheritance patterns on early heart development. Therefore, comprehensive genetic analysis in these patients is an essential way of predicting the prognosis and recurrence risk in families and ultimately improving patients’ quality of life due to better therapeutic options.

In this review, we examine the different types of variants and genes of different molecular genetics techniques to assess the diagnostic yield in different CHD sub-phenotypes. Given the complex inheritance pattern observed in CHD, we also consider possible future methods and frameworks to improve diagnostics and allow for better genotype-phenotype correlation in this patient group. Predicting recurrence risk and prognosis in CHD patients will ultimately allow for better treatment and lifelong therapeutic outcomes for CHD patients.

## Introduction

**Figure 1:**
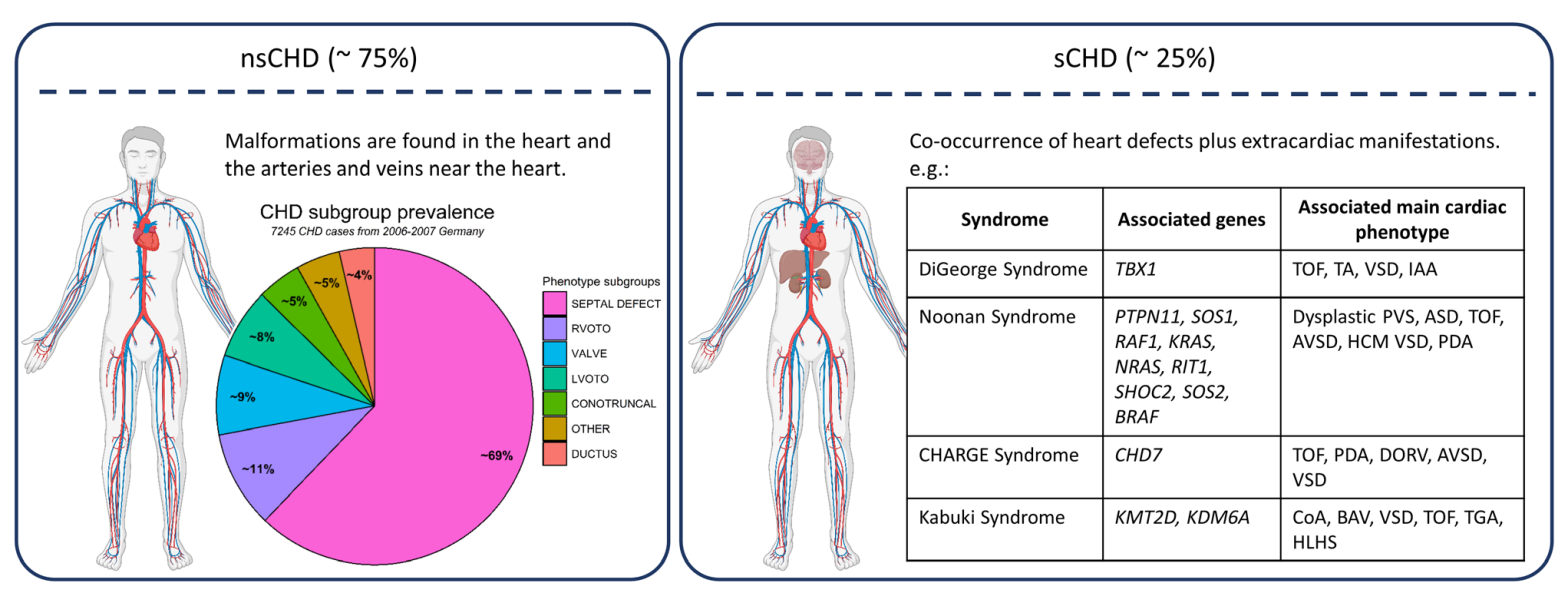
**Left:** Overview of isolated (non-syndromic) CHD (nsCHD). The most frequently observed CHD-subgroup of patients born with CHD from 2006–2007 in Germany [15]. SEPTAL DEFECT: ventricular septal defect (VSD), atrial septal defect (ASD), atrioventricular septal defect (AVSD); RVOTO (right ventricular outflow tract obstruction): total anomalous pulmonary venous connection (TAPVC), partial anomalous pulmonary venous connection (PAPVC), pulmonary stenosis (PS), pulmonary stenosis + ventricular septal defect (PA+VSD), pulmonary stenosis with intact ventricular septal (PA+IVS), truncus arteriosus communis (TAC), Tetralogy of Fallot (TOF); VALVE: aortic stenosis (AS), pulmonary stenosis (PS), Ebstein anomaly; LVOTO (left ventricular outflow tract obstruction): coarctation of the aorta (CoA), hypoplastic left heart syndrome (HLHS), aortic stenosis (AS), interrupted aortic arch (IAA); CONOTRUNCAL: Tetralogy of Fallot (TOF), dextro transposition of the great arteries (D-TGA), looped transposition of the great arteries (L-TGA), double outlet right ventricle (DORV), truncus arteriosus communis (TAC); OTHER: various CHD phenotypes; DUCTUS: patent ductus arteriosus (PDA). **Right:** examples of frequent syndromic heart defect (sCHD) and associated genes. sCHD includes various syndromes associated with heart defects and other extracardiac diseases and developmental disorders. TA = truncus arteriosus, dysplastic PVS = pulmonary valve stenosis, HCM = hypertrophic cardiomyopathy, BAV = bicuspid aortic valve, TGA = transposition of great arteries. Created with the use of BioRender.com and R (version 4.4.1).

Congenital heart defect (CHD) is one of the most common birth defects and affects approximately 1 %–2 % of all liveborn children, with a similar prevalence worldwide [1–3]. CHD comprises structural malformations of the heart and the great vessels and is often accompanied by life-threatening consequences [3]. Depending on the type and severity of the heart defect, patients may require early invasive procedures and lifelong follow-up due to both primary and secondary complications and extracardiac anomalies [4]. Primary complications can manifest as various symptoms, such as an increased susceptibility to infections or reduced physical performance. Secondary complications, like arrhythmias, may arise from early or late interventions during follow-up [5–7]. Primary and secondary complications are not only observed among non-syndromic CHD (nsCHD) (~75 % of cases), but also occur more frequently among the group of syndromic CHD (sCHD) (~25 % of cases) [8], which often present with extracardiac malformations, developmental disorders, and mental health problems (**Figure 1**), due to underlying causes not confined to the heart [9,10]. Therefore, understanding the genetic underpinnings of CHD and its impact on early heart development and lifelong consequences are important factors that need to be improved. Although CHD generally leads to lower life expectancy compared to the general population, the impact is particularly pronounced for critical cases [11,12]. The overall influence on the healthcare system is substantial, especially considering that more than 90 % of affected individuals now reach adulthood due to advancements in surgical and medical treatments [13,14].

Current studies in unselected cohorts allow for a molecular diagnosis in 33 % to 46 % of all CHD patients with the help of sequencing (short read Exome Sequencing (srES) and/or short read Genome Sequencing (srGS)) [1,13,16]. Many known associations follow a mono-allelic inheritance pattern and often arise de novo. Approximately 20 % of the sCHD patients have a *de novo* variant [2,17]. Biallelic inheritance patterns, non-coding variants and polygenetic mechanisms are less well researched [17,18]. In addition, incomplete disease penetrance and a high inter- and intrafamilial variability of phenotypes are frequently observed and might be partially explained by gene-environment interactions [2,18], which include but are not limited to pre-gestational diabetes, maternal iron deficiency, and increased maternal age [19–21]. It is assumed that around 10 % of cases are caused by environmental factors [18].

Therefore, the adoption of different sequencing techniques in combination with in-depth phenotyping, which would include early factors during pregnancy, will not only further shed light on those patients with a diagnosis by improving the genotype-phenotype correlation but also improve our understanding of the currently unexplained approximately 50 % of patients [17,22].

## Known genetic causes of CHD

The aetiology of CHD is complex and incompletely understood. CHD is a multifactorial disease caused by environmental factors and genetics [2,17]. Known genetic causes of congenital heart defects include aneuploidies (abnormal number of chromosomes), Copy Number Variants (CNVs), Single Nucleotide Variants (SNVs) and small Insertions and Deletions (InDels) [2]. These variants often exert their function by disturbing the precise regulation of cell-cell communication, which is essential for human embryogenesis and incredibly complex organ development, such as the heart [2,23–25]. Various important heart signalling pathways overlap, and interactions, feedback loops and crosstalk increase the complexity of these regulatory processes. Several well conserved and established transcription factors, like members of the TBX- (e.g. *TBX5*) and GATA-family (e.g *GATA4*) are important regulators of early cardiogenesis. These transcription factors and involved signalling pathways like TGF-β, WNT and Notch not only restrict their function on early heart development, but also coordinate maturation. Heart maturation includes different processes like energy metabolic, structural and contractile maturation (reviewed in detail Zubrzycki et al. 2024, Sakamoto and Kelly 2024) [26,27]. These aspects are further modulated by established links between CHD and cilia-mediated cell signalling [28].

**Figure 2:**
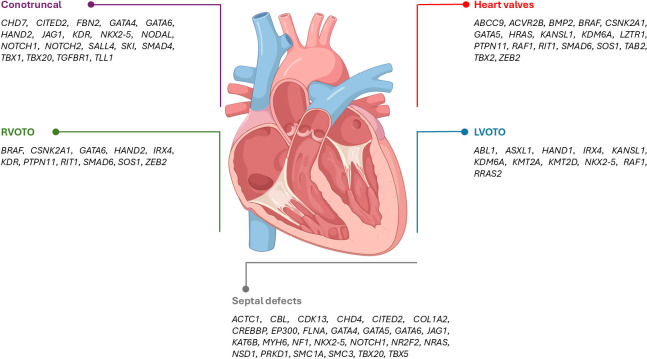
Overview of CHD-associated genes for which a predominance of certain CHD-subtypes is observed based on unpublished internal data or previous publications [29–31]. An extended list of CHD-related genes can be found in **Table 1**. Created with the use of BioRender.com. LVOTO: left ventricular outflow tract obstruction, RVOTO: right ventricular outflow tract obstruction.

The genes involved in these critical aspects of heart development impact different types of CHD-subgroups. **Figure 2** represents the predominant subgroup-associations of human CHD genes based on developmental knowledge reviewed by Xie et al. (2023) [29], Morton et al. (2022) [30] and more recent evidence from a meta-analysis [31] combined with careful evaluation of known human disease association.

The detection of the different types of variants was closely linked to the advancement of various molecular techniques of detection, starting with karyotyping and fluorescence in-situ hybridisation (FISH) to visualise and detect larger structural rearrangements and aneuploidies [32–34]. Aneuploidies represent the first identified genetic cause of CHD. They are commonly diagnosed in patients with sCHD due to a large number of involved genes and subsequent complex systemic effects [17]. Aneuploidies are found in approximately 8 % to 10 % of patients with CHD [2]. One typical example are patients with trisomy of chromosome 21, from which around 40 % to 50 % present with a septal defect (VSD, ASD or AVSD) [2,35].

Comparative genomic hybridisation arrays (CGH-array) or chromosomal microarrays (CMA) lead to the identification of CNVs in patients with sCHD and nsCHD [36]. Again, larger CNVs involving multiple genes correlate with a syndromic phenotype [2]. Pathogenic CNVs are a genetic cause in approximately 3 %–25 % of sCHD cases and also in 3 %–10 % of nsCHD cases [2]. CNVs in several genomic hotspots are frequently enriched in CHD cohorts, including 1q21.1 (duplication and deletion; TOF, LVOTO and septal defects), 2q13 (deletion; septal defects and aortic coarctation), 7q11.23 (Williams–Beuren syndrome; deletion; supravalvular aortic stenosis), 8p23.1 (duplication and deletion; AVSD, PVS, VSD), 11q24 (deletion; LVOTO), 15q11.2 (deletion; anomalous pulmonary venous connection, LVOTO, septal defects and PDA), 16p11.2 (duplication and deletion, TOF and single ventricle), and 22q11.2 (Velocardiofacial syndrome, duplication and deletion, TOF, LVOTO and septal defects) [2,37,38]. They often affect dosage-sensitive cardiac transcriptional factors [2].

The diagnostic yield for CHD by srES and srGS is estimated to be around 34 % [1,39]. Approximately 10 % of these cases show a *de novo* disease-causing SNV [39,40]. Typically, SNVs are found in genes involved in the embryonic development of the heart and encoding transcription factors, proteins in signalling pathways, genes related to histone modification, cilia or structural components of the heart [2]. srES has been successfully applied in numerous studies leading to revelation of specific *de novo* and inherited variants in novel and known genes associated with CHD [9,41–43]. Despite the selective detection of variants, srES can answer up to 11.5 % in isolated and ~15 % in sCHD cases [44] missing deeper intronic or regulatory regions [45]. In contrast, srGS provides coverage for both coding and non-coding regions, considerably extending the scope of analysis and leading to a higher diagnostic yield [1,46]. Slavotinek et al. (2024) showed a significant increase in the diagnostic rate for srGS (41.4 %) compared to srES (24.6 %) in cases with sCHD [1]. Additionally, it has been shown that srES-negative cases can benefit from srGS [46]. These aspects argue for srGS as a more suited diagnostic tool in future studies. Despite this, srGS is also associated with high demands on bioinformatic analytical power, data storage capacities, and higher costs [47]. Another challenge is variant classification and interpretation [46]. It is assumed that rare coding variants can explain approximately 45 % of CHD cases [48]. Studies have shown that non-coding variants are also involved in CHD pathogenesis. However, these variants need to be researched further in order to be able to make reliable statements [49].

**Table 1:**
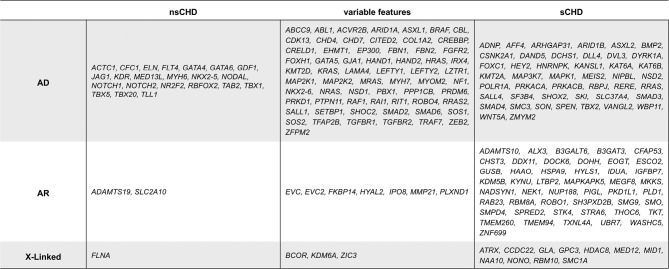
List of genes with high evidence for an association with CHD development. Genes are sorted according to observed inheritance patterns and isolated and syndromic heart defects. AD = autosomal dominant, AR = autosomal recessive, nsCHD = non-syndromic CHD, sCHD = syndromic CHD.

Although their impact on gene expression and gene regulation is well documented [50,51], confirmatory studies in suitable models are often needed to provide causative evidence [48,52]. Currently, less studied causes of CHD include somatic mosaic variants in approximately 1 % of cases [53,54] and polygenic risk scores obtained from large genome-wide association studies (GWAS). Thus far, only a few studies have explored the contribution of common genetic variants to CHD and achieving sufficient statistical power remains challenging [55–57].

As mentioned above, CHD is a multifactorial disease and is caused by genetic and environmental factors. However, due to a lack of experimental approaches, it is hard to analyse oligogenic or polygenic inheritance leading to complex phenotypes [58]. But different studies showed that a complex CHD-phenotype could be explored looking at digenic and oligogenic inheritance [58–60].

## Commonly CHD associated genes and their clinical impact

The Human Phenotype Ontology (HPO) term “Abnormal heart morphology” (HP:0001627) is associated with more than 1559 genes, many of which do not show sufficient evidence for an association with human CHD [61]. Given the high variation regarding a CHD consensus panel list, a thorough manually curated list of CHD-associated genes from different resources (panelapp.genomicsengland.co.uk/, www.ebi.ac.uk/, gene2phenotype/g2p_vep_plugin, clinicalgenome.org/) and an in-house dataset derived from large population datasets [31,62] gives a good representation of genes to be examined in the context of CHD (**Table 1**).

CHD genes can be divided into those associated with nsCHD or sCHD. Of note, this separation should be considered cautiously, as many CHD genes are linked to a spectrum of phenotypic outcomes ranging from isolated CHD to subtle extracardiac manifestations to syndromic CHD. A considerable subset of genes cannot be assigned to either end of this spectrum, herein termed variable features (**Table 1**). Also, genes listed under nsCHD may be associated with isolated CHD in some cases and a syndromic condition in others. A well-studied example is the *NOTCH1*-gene, reported in isolated CHD and the syndromic context of the Adams-Oliver syndrome [63–65]. Taking these aspects into account, broader gene testing seems to be advisable even in cases of isolated heart defects.

The diagnostic yield is higher in sCHD patients compared to nsCHD patients and higher when applying srGS compared to srES analysis [1]. The yield after a srES is approximately 11 %–15 % [44]. In a selected cohort of critically ill infants (<1 year) with structural CHD, srGS can reach levels of up to 46 % [16]. Another significant increase of srGS over srES of ca. 14 % was found by Bertoli-Avella et al. (2021) [46]. The increase in diagnostic yield can be explained by captured non-coding variants and CNVs, which are missed by srES [46,47].

Due to this and the decreasing NGS costs [66], srGS might become the method of choice [16]. Knowing the genetic cause can benefit the patient and the clinician [67]. Firstly, it allows risk assessment of affected individuals’ future offspring and enables informed decisions concerning family planning [67]. However, since CHD is usually diagnosed during the second trimester and given the phenotypic variability even among well-described causes, decisions concerning pregnancy termination are challenging to make [67]. Secondly, early identification of CHD causes of causative variants will allow for risk stratification, enabling pre-emptive management of expected co-morbidities and informing the risk of postoperative complications and mortality [67].

For example, patients with a single ventricle correlate with larger CNVs associated with adverse effects on growth and neurodevelopmental outcomes [68]. Furthermore, the length of CNVs and *de novo* mutations are negatively related to post-surgical outcomes in CHD cases [69]. In contrast, CNVs in CHD patients were significantly linked to decreased transplant-free survival [70]. Similarly, an early diagnosis in CHD patients will help to evaluate patients with heterotaxy for primary ciliary dyskinesia and causative variants in hypoplastic left heart syndrome (HLHS) patients, which are both associated with a worsening prognosis [71–74]. More importantly, recent evidence in large CHD cohorts also points to an increased risk of cancer and a higher prevalence of immunodeficiency [75,76].

## Future aspects

Given the heterogeneous nature of CHD, which requires testing of broad gene lists, variants of unknown significance are a frequent challenge for determining the genetic cause of the disease. Leveraging bulk and single-cell cardiac datasets will not only help to better detect and characterise disease variants [77], but also suggest novel candidate genes in CHD [78]. Moreover, an approach that increasingly demonstrates its value in a clinical diagnostic context is the evaluation of epigenetic markers, such as DNA methylation (DNAm) [79], also referred to as epi-signatures. These DNAm epi-signatures are valuable for diagnostic screening and reclassifying ambiguous genetic findings. So far, several epi-signatures, including syndromes associated with CHD, such as the microdeletion of 7q11.23 (Williams–Beuren syndrome) and 22q11.2 (Velocardiofacial syndrome), have been identified and subsequently integrated into clinical diagnostics [80].

The introduction of long-read sequencing (lrGS) might offer the detection of previously undetectable variants such as larger and complex structural variants and repetitive sequences in addition to enabling combined variant phasing, distinguishing highly homologous genomic areas as well as detecting epigenetic modifications such as DNAm [45,81]. Although lrGS is still costly, third-generation sequencing will help overcome the limitations of short-read sequencing data [81].

Novel found candidate genes need experimental validation to be confirmed as CHD causative genes. The experimental setup might look different depending on the nature and function of the gene. Morphological and physiological cardiac changes can be studied using *in vivo* and *in vitro* models of CHD, most often utilising stem cell-derived cardiomyocytes. A study performed on human induced pluripotent stem cells (hiPSCs) from patients with pulmonary atresia revealed a decrease in gene expression of components involved in the cardiac contractile apparatus, providing a molecular explanation for the underlying pathomechanism [61]. Such approaches can help to investigate the role of found variants. Hao et al. (2022) observed cardiac abnormalities in *in vivo* CHD models, and the transcriptional effects of these changes were analysed through massively parallel reporter assays (MPRAs), which looked at enhancer activities [83]. A recent publication also looked at non-coding *de novo* variants and assessed their role using assays to analyse the effect on transcription factors or repressors binding [48]. Similarly, Kathiriya et al. (2021) utilised stem cell-derived cardiomyocytes to model *TBX5* haploinsufficiency, while Paige et al. (2021) generated hiPSCs from patients with HLHS to investigate its pathogenesis [84,85]. Additionally, hiPSCs can be employed for single-cell transcriptome analyses.

Despite their usefulness, hiPSCs are often utilised in a two-dimensional format, which does not effectively replicate the complex three-dimensional architecture of human cardiac tissue. To address this limitation, self-assembling heart organoids can be generated from hiPSCs, and in 2021, cardiac organoids were employed to model and study congenital heart disease induced by pregestational diabetes [86].

The combination of advances in sequencing technologies using both genomic DNA and single-cell transcriptomics combined with advanced modelling approaches, such as human cardioids, will improve insight into disease causation and novel therapeutic options for CHD patients. Combined with future approaches using deep learning imaging analysis [87] and clinical genomic AI-based algorithms [88] or the combination of both, patients and especially the growing group of adult CHD cases [89], will be able to significantly benefit from advances in risk stratification and personalised clinical management.
